# Impact of Flexible Communication Skills Training on Healthcare Professionals’ Communication Knowledge and Confidence

**DOI:** 10.21203/rs.3.rs-6864165/v1

**Published:** 2025-08-22

**Authors:** Gabriel Hooper, Mindy J. Vanderloo, Kristin Akers, Emma Braun, Megan Call, Claire Ciarkowski

**Affiliations:** University of Utah; University of Utah Health; University of Utah Health; University of Utah; University of Utah Health; University of Utah Health

**Keywords:** communication skills training, VitalTalk, Communication confidence, health care professionals

## Abstract

**Background:**

Effective communication is critical to high-quality patient care, yet healthcare professionals often feel unprepared for complex conversations. This quality improvement study evaluated whether a flexible communication training program could enhance HCP knowledge and confidence across professional roles.

**Method:**

Adapted from VitalTalk^™^, the curriculum included interactive lectures, skills practice with trained actors, and peer/facilitator feedback, delivered virtually or in person.

**Results:**

A total of 102 HCPs enrolled; 64 (63%) completed pre-, post-, and 1-month follow-up surveys. Knowledge and confidence scores improved significantly post-training and were largely sustained at 1-month. Physicians reported higher confidence than nurses and advanced practice providers (APPs), though knowledge scores did not differ by profession. Confidence in addressing emotion was retained at follow-up, while confidence in quickly establishing trust declined.

**Conclusion:**

These findings suggest that brief, flexible communication training improves HCP confidence and knowledge, though ongoing practice may be needed to maintain gains. This model may support interprofessional training with minimal disruption to clinical schedules.

## Introduction

Communication is foundational to the patient-healthcare professional relationship. Effective communication is associated with improved patient adherence, satisfaction, and health.^1,2,3^ Despite the availability of training courses, healthcare professionals (HCP) continue to report feeling unprepared for difficult and complex communication.^4^ Time and accessibility are barriers to attending trainings, underscoring the need for flexible training courses.^5^ In this quality improvement study, we aimed to assess if flexible communication training could improve HCP communication knowledge and confidence across professional healthcare roles.

## Methods

This training was adapted from VitalTalk^™^ and included: 1) brief interactive lectures, 2) skills practice with trained actors, and 3) real-time peer and facilitator feedback. Content focused on: responding to emotion, conflict, disclosing error, and delivering serious news. Six communication classes were held between 10/2022 and 11/2023. Each class enrolled a HCP cohort who attended 2 to 3 sessions for a total of 4 to 6 training hours. Prioritizing flexibility, the course was offered virtually and in person. Participants were surveyed on their knowledge and confidence in communication skills prior to the course, at course completion, and 1 month after the course. Items were scored on a five-point Likert scale of Strongly Disagree (1) to Strongly Agree (5). Composite knowledge and confidence scores were constructed by averaging 6–7 questions in each category, where 5 is a high score. Questions related to establishing rapport and addressing emotion were compared using Wilcoxon signed rank test and a linear mixed effect model, respectively. Linear mixed effect models were used to assess differences by profession. This study was acknowledged by the University of Utah IRB (#00134460).

## Results

In total, 102 HCP enrolled in the classes and 64 (63%) participated in 121 surveys (58 pre-, 40 post-, 23 1-month-post surveys). Surveys were completed by 28 physicians, 12 Advanced Practice Providers (APPs), 10 nurses, and 14 missing or ‘other’ healthcare professionals (e.g., support staff, supervisors, coordinators, chaplains, etc.).

Knowledge and confidence scores across all HCPs improved significantly post-course and at 1-month (Table 1). There were no significant differences in knowledge between HCP types (Table 1). Across all surveys, physicians reported higher confidence compared to nurses and APPs (Table 1).

**Figure 1:**
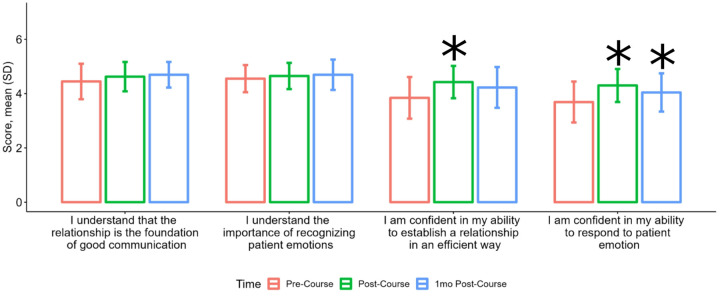
Change in knowledge and confidence in establishing rapport quickly and responding to emotion. Knowledge was assessed using Wilcoxon signed rank test comparing pre and post-course, but there was not sufficient data to assess pre-course to 1 month post-course for these knowledge questions. Confidence questions were analyzed using a linear mixed effect model. * = p value <0.05 compared to pre-course score.

On the pre-course survey, most participants endorsed knowing the importance of establishing relationships (mean 4.4, SD 0.7) and recognizing emotions (4.6, SD 0.5); however, confidence in establishing rapport and respond to emotion was lower [3.8 (SD 0.8) and 3.7 (SD 0.8)] (Fig. 1). Confidence in these skills improved significantly at course completion (Fig. 1). HCP reported sustained improvement in confidence to address emotion after 1 month; however, confidence to establish trust quickly was not maintained (Fig. 1).

## Discussion

Our study demonstrates training with flexible time and travel commitments meets the needs of HCPs and improves knowledge and confidence in communication skills. While knowledge of communication skills was as high across all HCP types, confidence in these skills was lower in nursing and other staff when compared to physicians and APPs. This gap in confidence between professions may be related to differences in training.^6^

Interestingly, prior to the course, most HCPs recognized the importance of foundational communication skills yet endorsed lower confidence in using these skills. All participants, regardless of profession, reported improvement in confidence following the course. Notably, confidence in responding to emotion but not in establishing trust was retained after 1 month suggesting follow up trainings or skills practice may be needed to maintain confidence across skills. As skills practice is vital to change behavior, we do believe meaningful training with small, repeated practice can create a lasting impact.

This study has several limitations. First, this is a small cohort and survey participation rates waned overtime. Second, 1-month follow up surveys may not capture durable change in communication confidence. Last, confidence may not be a representative surrogate of practice change or impact on patient outcomes.

In conclusion, a brief in-person or virtual communication training can effectively improve knowledge and confidence in communication skills across a variety of HCPs; however, follow-up training may be necessary to maintain these skills and the confidence to use them. Further study is planned on the effectiveness of flexible interprofessional health care communication training, particularly longitudinally and how the significant findings of this training can be translated to clinical practice and patient-centered outcomes.

## Figures and Tables

**Table 1:** Comparison of Knowledge and Confidence over time and between HCP types.

Category	Variable	Comparison	Estimated Mean Difference (95% CI)	p-value
Knowledge	Time	(Post-Course) - (Pre-Course)	0.431 (0.276, 0.585)	< 0.001
		(1mo Post-Course) - (PreCourse)	0.337 (0.131, 0.542)	0.002
		(1mo Post-Course) - (PostCourse)	−0.094 (−0.309, 0.121)	0.386
	Profession*	APP - Nurse	0.304 (−0.23, 0.838)	0.439
		MD - Nurse	0.461 (−0.017, 0.94)	0.063
		MD - APP	0.158 (−0.247, 0.562)	0.729
		Other - Nurse	0.294 (−0.222, 0.81)	0.434
		Other - APP	−0.01 (−0.526, 0.507)	> 0.999
		Other - MD	−0.167 (−0.618, 0.283)	0.754
	Leadership	Yes - No	0.495 (0.167, 0.823)	0.004
Confidence	Time	(Post-Course) - (Pre-Course)	0.588 (0.425, 0.751)	< 0.001
		(1mo Post-Course) - (Pre-Course)	0.355 (0.138, 0.573)	0.002
		(1mo Post-Course) - (Post-Course)	−0.232 (−0.459, −0.006)	0.045
	Profession*	APP - Nurse	0.252 (−0.294, 0.799)	0.358
		MD - Nurse	0.691 (0.203, 1.18)	0.006
		MD - APP	0.439 (0.023, 0.855)	0.039
		Other - Nurse	0.491 (−0.045, 1.026)	0.071
		Other - APP	0.238 (−0.299, 0.776)	0.377
		Other - MD	−0.201 (−0.67, 0.269)	0.394
	Leadership	Yes - No	0.497 (0.051, 0.942)	0.030

1 To adjust for multiple comparisons, a Tukey adjustment for a family of 4 estimates was used when computing p-values and confidence intervals for the pairwise comparisons between professions.

## Data Availability

The datasets generated during and/or analyzed during the current study are not available because of the potential exposure of sensitive employee information, particularly in small and unique hospital groups.
